# USF1 transcriptionally activates USP14 to drive atherosclerosis by promoting EndMT through NLRC5/Smad2/3 axis

**DOI:** 10.1186/s10020-024-00798-8

**Published:** 2024-02-29

**Authors:** Zhiwen Zhang, Quan Guo, Chao Ma, Zhenzhou Zhao, Qingbo Shi, Haosen Yu, Lixin Rao, Muwei Li

**Affiliations:** 1 Department of Cardiology, Zhengzhou University People’s Hospital, Henan Provincial People’s Hospital, Zhengzhou, Henan 450000 China; 2 Department of Cardiology, Central China Fuwai Hospital, Zhengzhou, Henan 450000 China

**Keywords:** Atherosclerosis, EndMT, USF1, USP14, NLRC5, Smad2/3

## Abstract

**Background:**

Endothelial-to-Mesenchymal Transformation (EndMT) plays key roles in endothelial dysfunction during the pathological progression of atherosclerosis; however, its detailed mechanism remains unclear. Herein, we explored the biological function and mechanisms of upstream stimulating factor 1 (USF1) in EndMT during atherosclerosis.

**Methods:**

The in vivo and in vitro atherosclerotic models were established in high fat diet-fed ApoE^−/−^ mice and ox-LDL-exposed human umbilical vein endothelial cells (HUVECs). The plaque formation, collagen and lipid deposition, and morphological changes in the aortic tissues were evaluated by hematoxylin and eosin (HE), Masson, Oil red O and Verhoeff-Van Gieson (EVG) staining, respectively. EndMT was determined by expression levels of EndMT-related proteins. Target molecule expression was detected by RT-qPCR and Western blotting. The release of pro-inflammatory cytokines was measured by ELISA. Migration of HUVECs was detected by transwell and scratch assays. Molecular mechanism was investigated by dual-luciferase reporter assay, ChIP, and Co-IP assays.

**Results:**

USF1 was up-regulated in atherosclerosis patients. USF1 knockdown inhibited EndMT by up-regulating CD31 and VE-Cadherin, while down-regulating α-SMA and vimentin, thereby repressing inflammation, and migration in ox-LDL-exposed HUVECs. In addition, USF1 transcriptionally activated ubiquitin-specific protease 14 (USP14), which promoted de-ubiquitination and up-regulation of NLR Family CARD Domain Containing 5 (NLRC5) and subsequent Smad2/3 pathway activation. The inhibitory effect of sh-USF1 or sh-USP14 on EndMT was partly reversed by USP14 or NLRC5 overexpression. Finally, USF1 knockdown delayed atherosclerosis progression via inhibiting EndMT in mice.

**Conclusion:**

Our findings indicate the contribution of the USF1/USP14/NLRC5 axis to atherosclerosis development via promoting EndMT, which provide effective therapeutic targets.

**Supplementary Information:**

The online version contains supplementary material available at 10.1186/s10020-024-00798-8.

## Introduction

Atherosclerosis is a cardiovascular disorder featured by formation of plaques in the thickened artery wall, which remains a major driver of death and disablement (Libby et al. 2019). Rupture of unstable plaques may lead to thrombosis and blood flow interruption. Endothelial to mesenchymal transition (EndMT) has been identified to participate in the pathological development of atherosclerosis (Wesseling et al. 2018). EndMT facilitates atherosclerosis progression through promoting the secretion of pro-inflammatory molecules extracellular matrix protein synthesis that contributes to plaque buildup (Ross 1999). Previous studies have reported that pro-inflammatory molecule IL-1β and ox-LDL can induce EndMT, thereby promoting the progression of atherosclerosis (Maleszewska et al. 2013; Yoshimatsu et al. 2020). Given the important contribution of EndMT to atherosclerosis, suppression of EndMT represents an effective therapeutic strategy for atherosclerosis.

Upstream stimulating factor 1 (USF1) belongs to the basic helix-loop-helix leucine zipper family. USF1 inhibition has been demonstrated to ameliorate hyperlipidemia, adiposity, and atherosclerosis in mice, suggesting USF1 as a potential therapeutic target for atherosclerosis (Laurila et al. 2016). More importantly, as a key transcription factor, USF1 can modulate the transcription and expression of target genes via binding to the E-box motif (Ikeda et al. 2014). USF1 has been documented to regulate the transcription of genes that participate in glucose and lipid metabolism (Kristiansson et al. 2008; Plaisier et al. 2009). So far, the detailed mechanisms through which USF1 affects atherosclerosis progression are largely unknown, which deserve further investigation.

As an important protein degradation pathway, ubiquitination is involved in the pathogenesis of atherosclerosis. For instance, the loss of E3 ubiquitin ligase Peli1 led to inflammation and plaque destabilization during atherosclerosis development (Burger et al. 2022). Besides, another E3 ubiquitin ligase HRD1 could restrain ox-LDL-induced apoptosis of endothelial cells via increasing LOX-1 degradation (Li et al. 2020a, b). Ubiquitin specific protease (USP14) is one of deubiquitinating enzymes. Up-regulation of USP14 was found in atherosclerotic patients, and USP14 inhibition exerted an anti-atherosclerotic role in vitro (Liu et al. 2019). Of note, as predicted by JASPAR database, USF1 can directly bind to USP14 promoter, providing possibility that USF1 might influence atherosclerosis progression via transcriptional modulation of USP14.

NLR Family CARD Domain Containing 5 (NLRC5), a member of NLRs family, has been identified as a critical regulator of inflammation, innate immunity and fibrogenesis (Neerincx et al. 2013). A recent study found that NLRC5 delayed vascular remodeling through inhibiting smooth muscle cell dysfunction (Luan et al. 2019). NLRC5 knockout accelerated high fat diet-induced cardiac injury by promoting fibrosis and inflammation (Ma and Xie 2017). Particularly, NLRC5 deficiency could protect against myocardial fibrosis by repressing EndMT via inactivation of Smad2/3 pathway (Wang et al. 2020a, b). These findings suggest that NLRC5 might be influence atherosclerosis development through regulation of EndMT. More importantly, as predicted by Ubibrowser2.0 database, NLRC5 might be a potential target protein of USP14.

In this study, we demonstrated that USF1 transcriptionally activated USP14 to restrain NLRC5 degradation, which prompted atherosclerosis by facilitating EndMT by activation of Smad2/3 pathway. Our results elaborate the pathological mechanism of atherosclerosis and open a new approach for clinical practice.

## Materials and methods

### Clinical samples

Serum samples were collected from 50 patients with atherosclerosis from Zhengzhou University People’s Hospital, Henan Provincial People’s Hospital. Meanwhile, 50 serum samples were obtained from healthy volunteers and serve as normal control. The diagnosis of atherosclerosis was based on the value of the carotid intima-media thickness (CIMT) of the common carotid artery. Exclusion criteria were cardiovascular and cerebrovascular diseases, cancer, or infection. The blood samples were taken before any medications were given to the patients. Before sample collection, written informed consent was provided by all participants. Table 1 shows the clinical characteristics of the patients, and their association with atherosclerosis. This study was approved by the Ethics Committee of Zhengzhou University People’s Hospital, Henan Provincial People’s Hospital.

**Table 1:** Clinical characteristics of subjects in the study

Characteristics of the cases	Normal (total 50)	Atherosclerosis (total 50)	*P* value
**Gender**			0.1609
male	22	30	
female	28	20	
**Age (years)**			0.2976
< 60	29	35	
> 60	21	15	
**History of smoking**			0.0969
YES	27	36	
NO	23	14	
**CKD** **(chronic kidney disease)**			0.6845
YES	19	22	
NO	31	28	
**Diabetes**			0.6820
YES	32	29	
NO	18	21	
**Body mass index**, **Kg/m2**			0.0023**
> 25	20	36	
< 25	30	14	

Note: **,*P* < 0.01; Chi-square test

### Animal model

Six-week-old male *ApoE*^*–/–*^ mice were purchased from Hunan SJA Co., Ltd (Changsha, China) and fed with a Western high fat diet (Hunan SJA Co., Ltd) for 12 weeks. For inactivation of Smad2/3 pathway, 4.2 mg/kg SB431542 (MedChemExpress, USA) was daily intraperitoneally injected into mice. DMSO was served as the vehicle control. Finally, all mice were sacrificed by cervical dislocation and the aortic tissues were obtained. The animal protocol was approved by the Ethics Committee of Zhengzhou University People’s Hospital, Henan Provincial People’s Hospital.

### Cell culture and treatment

Human umbilical vein endothelial cells (HUVECs) were purchased from American Type Culture Collection (USA) and cultured in endothelial growth medium (ScienCell, USA) supplemented with 5% FBS (Gibco, USA) and endothelial growth factors. The confluent HUVECs at passages 6–7 were maintained in basal medium containing 0.5% FBS for 8 h, followed by stimulation with ox-LDL (100 µg/mL) for 24 h to induce EndMT (Diao et al. 2022; Gong et al. 2019; Qiu et al. 2016). Thereafter, the cells were collected for further experiments.

### Cell transfection

HUVECs were transfected with shRNA targeting USF1 (sh-USF1), sh-USP14, USF1 overexpression plasmid (oe-USF1), oe-USP14, oe-NLRC5, or corresponding negative controls (sh-NC, oe-NC) using Lipofectamine 2000 (Thermo Fisher, USA). All shRNAs or plasmids were provided by GenePharma.

### Quantitative real time polymerase chain reaction (RT-qPCR)

Total RNA was isolated from human serum samples, HUVECs, and aortas using TRIzol reagent (Thermo Fisher) and reverse-transcribed into cDNA using the RT Master Mix for qPCR II (MCE, USA). RT-qPCR was performed using the Super Real Premix Plus Kit (Tiangen, Beijing, China). The levels of genes normalized to GAPDH were calculated using the 2^−ΔΔCT^ method. Table 2 shows the primer sequences.

**Table 2:** Oligonucleotide primer sets for RT-qPCR

Name	Sequence (5’¬3’)	Length
USF1 F	CACTAAACTCTGGGGCTTGTCC	22
USF1 R	CACCAGCCACTGCTAAACATCC	22
USP14 F	GAGTTGGACCTTT-CCAGA	19
USP14 R	TGCTTGCACAG-ATGTGA	18
CD31 F	TCAAGCCTCAGCACCAGA	18
CD31 R	GCACTCCTTCCACCAACAC	19
α-SMA F	CCTGAAGAGCATCCCACCCT	20
α-SMA R	ACCATCTCCAGAGTCCAGCACG	22
NLRC5 F	TGAGGGAGTCTGCACTATGGA	21
NLRC5 R	TCCGATTCAGGGCTCAGGTA	20
GAPDH F	GGGAGCCAAAAGGGTCAT	18
GAPDH R	GAGTCCTTCCACGATACCAA	20

### Western blotting

Protein samples were extracted using the commercial extraction kit (Thermo Fisher). The protein lysates were subjected to SDS-PAGE and blotted onto polyvinylidene fluoride membranes. After blocking in 5% bovine serum albumin for 1 h, the membranes were incubated with the primary antibodies against CD31 (A19014, 1:1000, ABclonal, Wuhan, China), VE-Cadherin (bs-22363R, 1:500, Bioss, Beijing, China), α-SMA (A17910, 1:500, ABclonal), vimentin (A19607, 1:1000, ABclonal, Wuhan, China), USF1 (sc-390,027, 1:1000, Santa Cruz, USA), USP14 (A19589, 1:500, ABclonal), NLRC5 (sc-515,668, 1:1000, Santa Cruz), Ubiquitin (ab140601, 1:1000, Abcam, UK), Smad2 (A19114, 1:500, ABclonal), Smad3 (A19115, 1:500, ABclonal), p-Smad2 (AP0269, 1:500, ABclonal), p-Smad3 (AP0727, 1:500, ABclonal), β-actin (AC006, 1:1000, ABclonal) overnight at 4 °C. Subsequently, the membranes were reacted with the secondary antibody (1:5000, ABclonal). The membranes were visualized using the Clarity Western ECL Substrate (BIO-RAD, USA).

### Immunofluorescence staining

The aortic tissues were fixed with 4% paraformaldehyde and cut into 4-µm sections, followed by permeabilization with 1% Triton X-100 and blocking with 5% BSA. For HUVECs, the cells with various treatments were fixed with 4% paraformaldehyde, infiltrated with 1% Triton X-100, and sealed with 5% BSA. Subsequently, the aortic sections or HUVECs were incubated with primary antibodies against CD31 (A19014, 1:50, ABclonal), α-SMA (A17910, 1:50, ABclonal) overnight at 4 °C. Then, FITC Goat Anti-Rabbit IgG (AS011, ABclonal) or Cy3 Goat Anti-Rabbit IgG (AS007, ABclonal) was applied. Finally, the sections or HUVECs were stained with DAPI, examined under a fluorescence microscope (Olympus, Japan) and quantified using the Image J software.

### Histopathological analysis

The collected aortic tissues were fixed in 4% paraformaldehyde, embedded in paraffin, and sectioned into 4 μm-sections. Subsequently, the sections were stained using the HE Staining Kit (Solarbio, Beijing, China) and Masson’s Trichrome Stain Kit (Solarbio) to evaluate atherosclerotic plaque formation and collagen accumulation, respectively. The sections were photographed under a light microscope.

### Enzyme linked immunosorbent assay (ELISA)

Serum samples were collected from the peripheral blood of mice. The cellular supernatant of HUVECs was collected. TNF-α, IL-1β, IL-6 levels in serum samples and cellular supernatant were assessed by ELISA using the commercial kits provided by Solarbio, according to the manufacturer’s instructions.

### Transwell assay

HUVECs were suspended in serum-free medium (2 × 10^5^ cells/mL). The upper chambers were added with 100 µL of cell suspension, while the bottom chambers were added with 800 µL of medium containing 10% FBS. After maintenance for 12 h, the unmigrated cells on upper chambers were wiped off. The migrated cells were fixed with methanol and stained with crystal violet. The photographs were taken and quantified using Image J software.

### Scratch assay

HUVECs were seeded into 12-well plates and cultured until confluence. A scratch was made using a pipette tip (500 µL), followed by washing with serum-free medium. At 0 and 48 h after scratch, HUVECs were photographed under a light microscope.

### Dual-luciferase reporter assay

The promoter sequences of USP14 were amplified and inserted into the pGL3 vector, named as wt-USP14. Mutant construct mut-USP14 was established by mutagenesis. The above luciferase reporter plasmids together with oe-USF1 or oe-NC were co-transfected into HUVECs using Lipofectamine 2000. The relative luciferase activity was detected using the Dual-Lucy Assay Kit (Solarbio) at 48 h after transfection.

### Co-immunoprecipitation (Co-IP)

HUVECs were lysed with the lysis buffer for IP (Beyotime, China) supplemented with PMSF and cocktail. Cell lysates were incubated with Protein A + G agarose beads at 4 °C for 1 h, followed by incubation with primary antibody against NLRC5 (sc-515,668, Santa Cruz) that was pre-conjugated in Protein A + G agarose beads overnight at 4 °C with rotation. Thereafter, the protein samples were eluted and determined by Western blotting.

### Chromatin immunoprecipitation (ChIP)

ChIP assay was performed using the ChIP kit (Abcam) following the manufacturer’s protocol. Briefly, HUVECs were cross-linked using 1% formaldehyde and then treated with glycine. Subsequently, ultrasonication was performed to break the chromatin. The chromatin fragments were precipitated with anti-IgG or anti-USF1 (Santa Cruz) at 4 °C. After treatment with NaCl and protease K, the DNA was purified and the enrichment of USP14 was measured by RT-qPCR.

### Oil red O staining

Plaque formation was evaluated by oil red O staining. Briefly, the aortic tissues were fixed in 4% paraformaldehyde and dehydrated with 15% and 30% sucrose, followed by embedding with optimal cutting temperature glue. The aortic tissues were sliced into 8-µm-slices and stained with Oil Red O solution (Solarbio) at 37 °C for 2 h. Under a light microscope, the images were taken and the plaque area was quantified according a previous study (Jiang et al. 2019).

### Verhoeff-Van Gieson (EVG) staining

To determine morphological changes, the sections of aortic tissues were deparaffinized and hydrated. The sections were immersed in EVG solution for 30 min, followed by incubation with ferric chloride differentiation solution until the background was grey white. Then, the sections were re-stained with VG solution. After dehydration using 100% ethanol, the sections were photographed under a light microscope.

### Statistical analysis

All values are expressed as mean ± standard deviation (SD) and analyzed by GraphPad Prism 8.0 software. For in vitro assay, both technical replicates and biological replicates were performed. For in vivo assay, biological replicates were performed. One-way analysis of variance (ANOVA) followed by Tukey’s post hoc test or Student’s t test wase performed to evaluate the differences between two groups or multiple groups. Correlation between USF1 and USP14 expression was analyzed by Pearson correlation analysis. *P* < 0.05 was considered statistically significant.

## Results

### Up-regulation of USF1 and USP14 in the serum of atherosclerotic patients

**Fig. 1:**
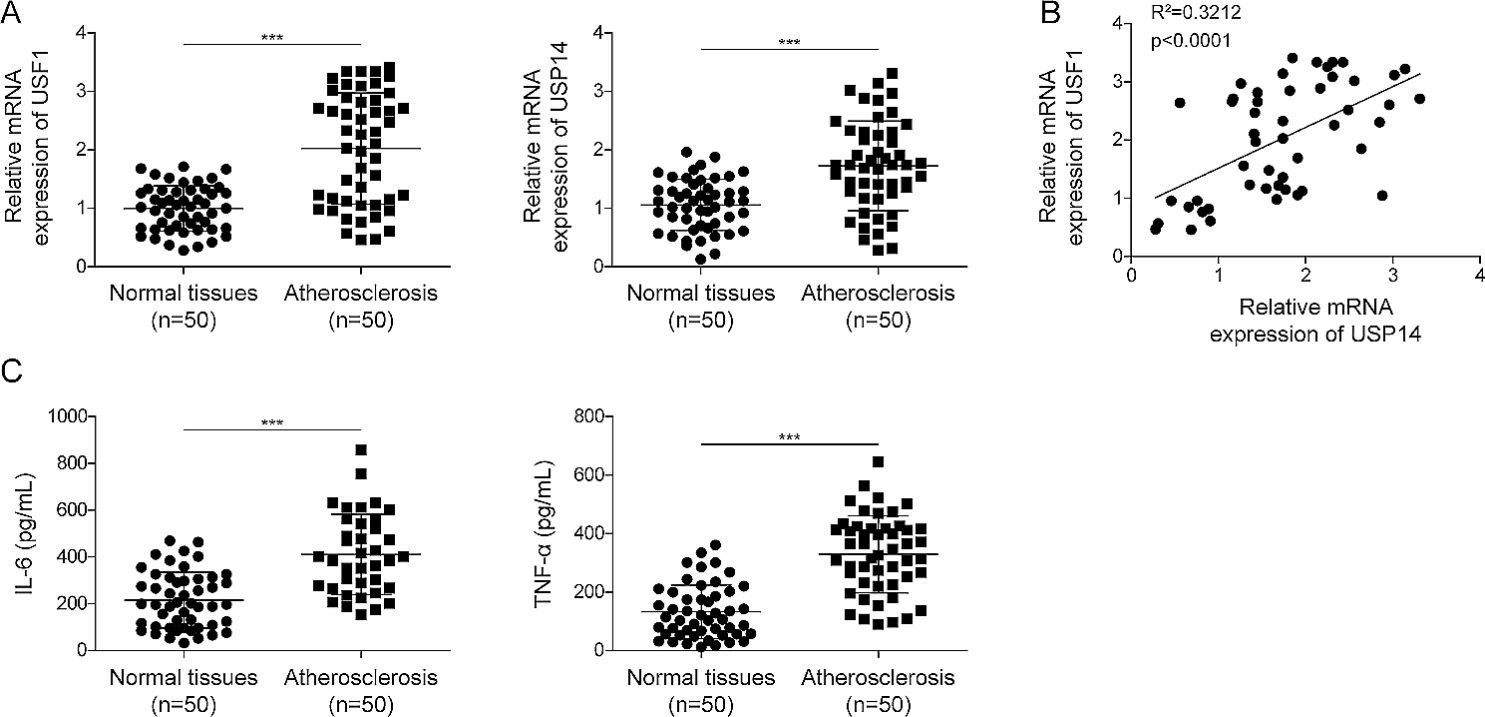
USF1 and USP14 were highly expressed in the serum of patients with atherosclerosis. (**A**) USF1 and USP14 expression was assessed by RT-qPCR in the serum samples of 50 patients with atherosclerosis and normal volunteers. (**B**) Pearson correlation analysis evaluated the correlation between USF1 and USP14 expression in patients with atherosclerosis. (**C**) The serum levels of inflammatory markers IL-6 and TNF-α were detected by ELISA. *n* = 50. ***p < 0.001

We first evaluated USF1 and USP14 expression in atherosclerotic patients. The mRNA levels of USF1 and USP14 were enhanced in the serum samples of atherosclerotic patients as compared with that in normal controls (Fig. 1A). Notably, USF1 level was positively correlated with USP14 level in atherosclerotic patients (Fig. 1B). The clinical characteristics of the patients are shown in Table 1. We found that atherosclerosis was closely correlated with body mass index, but not associated with age, gender, history of smoking, diabetics, or chronic kidney disease. Besides, the serum levels of IL-6 and TNF-α were significantly higher in patients with atherosclerosis as compared with normal volunteers (Fig. 1C). These results implied that dysregulation of USF1 and USP14 might contribute to atherosclerosis development.

### Development of EndMT in the atherosclerotic models

**Fig. 2:**
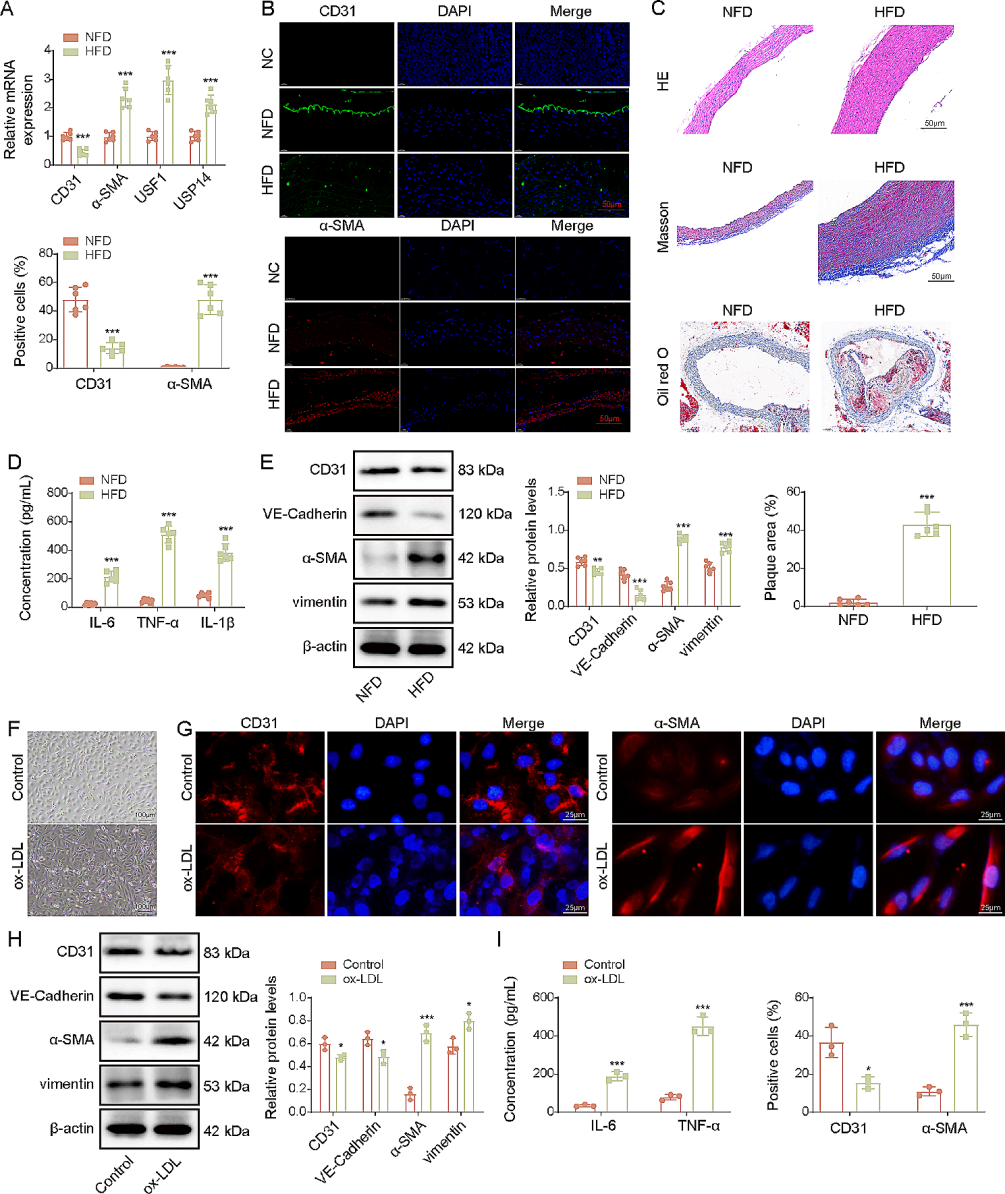
EndMT was induced in the in vivo and in vitro models of atherosclerosis. ApoE^–/–^ mice fed with high-fat diet was used as the mouse model of atherosclerosis. Normal chow-fed diet (NFD) represents normal mice. High-fat diet (HFD) represents atherosclerosis mice. (**A**) Expressions of CD31, α-SMA, USF1 and USP14 in aortas of ApoE^–/–^ mice were determined by RT-qPCR. (**B**) Immunofluorescence staining of CD31 and α-SMA in the aortas of ApoE^–/–^ mice (scale bar = 50 μm). (**C**) Atheromatous plaque formation, collagen accumulation, and lipid deposition were observed by HE, Masson, and Oil red O staining (scale bar = 50 μm). (**D**) IL-6, TNF-α and IL-1β levels in serum of mice were determined using ELISA kits. (**E**) Western blotting analysis of CD31, VE-Cadherin, α-SMA and vimentin levels in the aortas. ox-LDL-exposed HUVECs were used as the in vitro model of atherosclerosis. (**F**) The morphological changes of HUVECs were observed (scale bar = 100 μm). (**G**) CD31 and α-SMA expression in HUVECs was determined by immunofluorescence staining (scale bar = 25 μm). (**H**) Protein abundance of CD31, VE-Cadherin, α-SMA and vimentin in HUVECs was measured by Western blotting. (**I**) Concentrations of IL-6 and TNF-α in supernatant of HUVECs was detected using ELISA kits. For **A**-**E**, *n* = 6; for **G**-**I**, *n* = 3. *p < 0.05, **p < 0.01, ***p < 0.001

It has been recognized that EndMT is implicated in the pathological development of atherosclerosis (Huang et al. 2021). As compared with the normal mice, endothelial cell marker CD31 expression was lower, while mesenchymal cell marker α-SMA, and USF1 and USP14 levels were higher in the aorta of atherosclerotic mice (Fig. 2A&B). HE staining indicated serious vascular stenosis in *ApoE*^*–/–*^ mice (Fig. 2C). Masson staining showed excessive collagen deposition in the aortic tissues of mice with atherosclerosis (Fig. 2C). As determined by Oil red O staining, lipid deposition was found in atherosclerosis model group (Fig. 2C). The serum levels of IL-6, TNF-α and IL-1β were increased in atherosclerosis group (Fig. 2D). Besides, the levels of vimentin and α-SMA were higher, but the levels of CD31 and VE-Cadherin were lower in the aortic tissues of atherosclerotic mice than those in control mice (Fig. 2E). To establish the in vitro model of atherosclerosis, HUVECs were stimulated with ox-LDL. We found that ox-LDL treatment caused cellular morphological changes into spindle-like shape (Fig. 2F). Similarly, ox-LDL challenge reduced CD31, VE-Cadherin expression, but enhanced α-SMA and vimentin expression in HUVECs (Fig. 2G&H). Inflammation was triggered by ox-LDL as confirmed by increasing IL-6 and TNF-α production (Fig. 2I). These data indicated that EndMT was induced in atherosclerotic mice and ox-LDL-stimulated HUVECs.

### USF1 knockdown repressed EndMT in ox-LDL-stimulated HUVECs

**Fig. 3:**
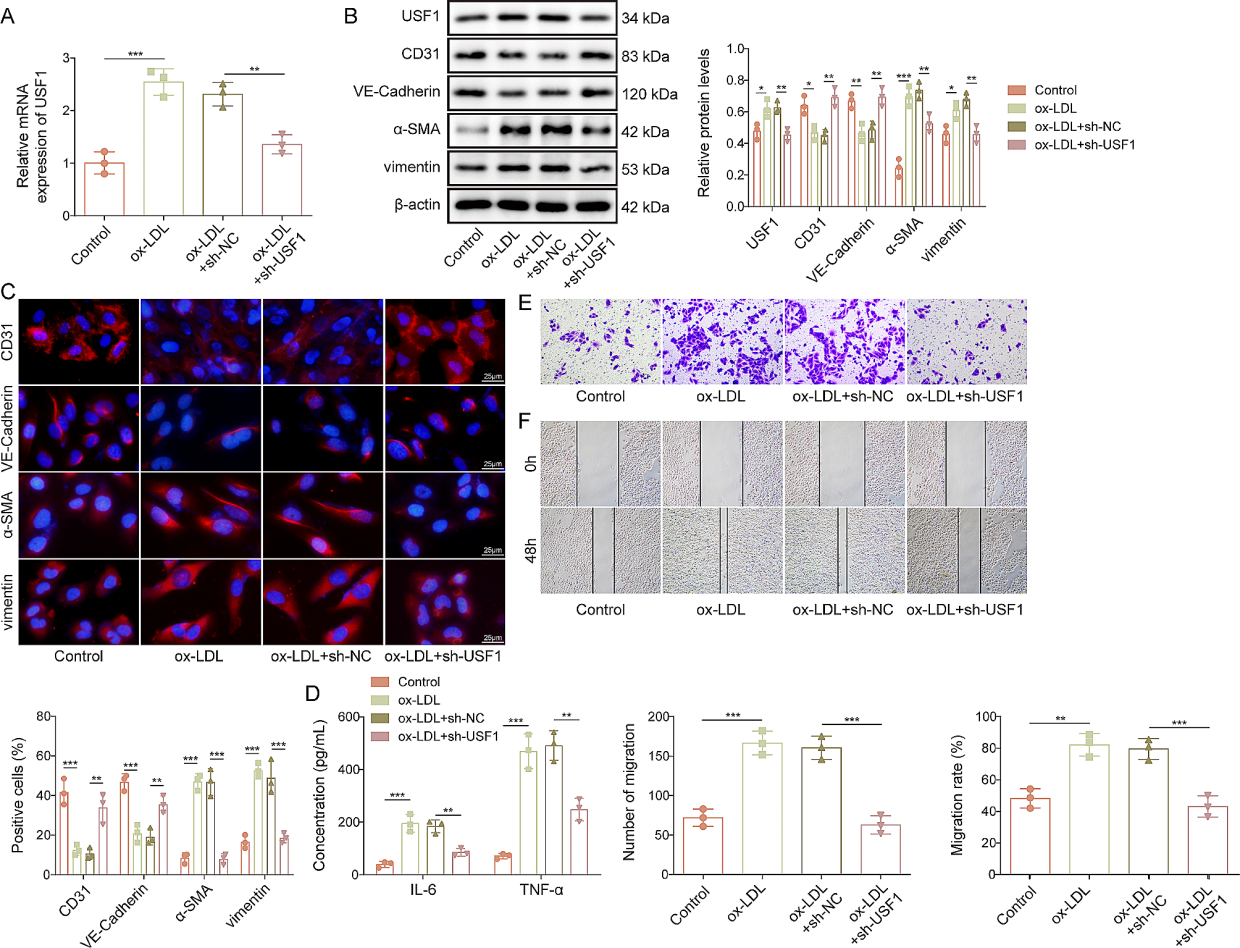
Ox-LDL-stimulated EndMT was restrained by USF1 down-regulation in HUVECs. sh-USF1-transfected HUVECs were treated with 100 µg/mL ox-LDL for 24 h. (**A**) USF1 mRNA level was evaluated by RT-qPCR. (**B**) Western blotting analysis of USF1, CD31, VE-Cadherin, α-SMA and vimentin levels in HUVECs. (**C**) Immunofluorescence staining of CD31, VE-Cadherin, α-SMA and vimentin in HUVECs (scale bar = 25 μm). (**D**) IL-6 and TNF-α production from HUVECs was assessed by ELISA. (**E**)&(**F**) Migration of HUVECs was assessed by transwell and scratch assays. *n* = 3. *p < 0.05, **p < 0.01, ***p < 0.001

To evaluate the biological function of USF1 in EndMT, we transfected sh-USF1 into HUVECs to knock down USF1 expression. We observed that ox-LDL-induced up-regulation of USF1 was reversed by sh-USF1 transfection (Fig. 3A&B). In addition, USF1 silencing effectively increased CD31 and VE-Cadherin expression, but decreased α-SMA and vimentin expression in ox-LDL-exposed HUVECs (Fig. 3B**&C**). Moreover, the release of IL-6 and TNF-α from HUVECs in the presence of ox-LDL was enhanced, which was weakened by USF1 deficiency (Fig. 3D). Furthermore, transwell and scratch assays indicated that USF1 down-regulation strikingly impaired migratory capability of ox-LDL-stimulated HUVECs (Fig. 3E**&F**). Based on these findings, we suggested that USF1 knockdown restrained ox-LDL-induced EndMT, inflammation, and migration in HUVECs in vitro.

### USF1 transcriptionally activated USP14 to promote NLRC5 de-ubiquitination

**Fig. 4:**
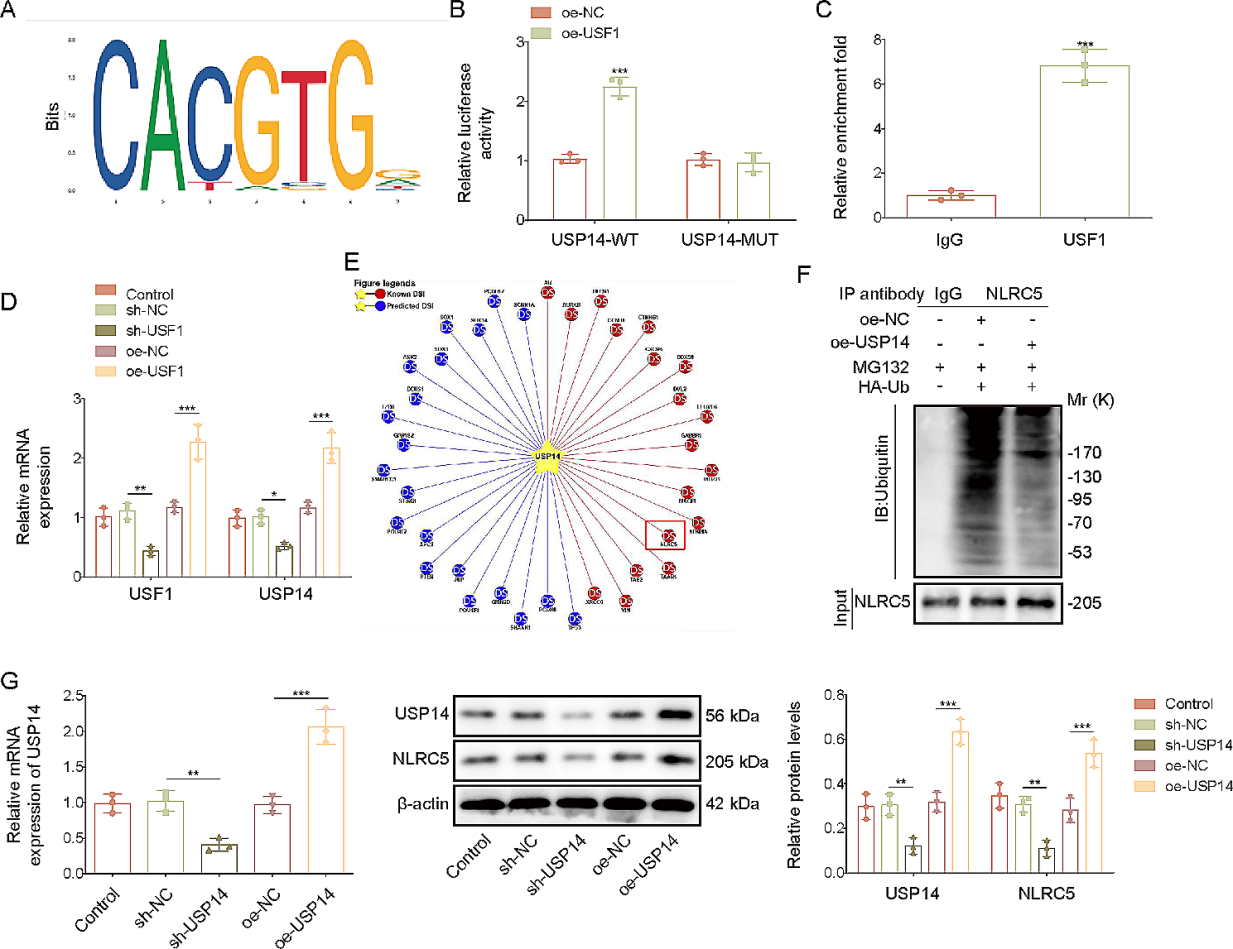
USF1 transcriptionally activated USP14 that caused NLRC5 de-ubiquitination. (**A**) Prediction of USF1 binding motif in the promoter of USP14 using the JASPAR database. (**B**) Relative luciferase activity in HUVECs transfected with luciferase plasmid constructs containing the USP14 promoter (wt and mut) in combination with USF1 overexpression plasmid. (**C**) ChIP assay confirmed the binding of USF1 to the USP14 promoter. (**D**) HUVECs were transfected with sh-USF1 or oe-USF1. USF1 and USP14 levels were measured by RT-qPCR. (**E**) Ubibrowser2.0 database predicted the potential de-ubiquitination targets of USP14. (**F**) Co-IP assay analysis of NLRC5 ubiquitination. (**G**) USP14 and NLRC5 levels were determined by RT-qPCR and Western blotting. *n* = 3. *p < 0.05, **p < 0.01, ***p < 0.001

To explore the modulatory mechanism through which USF1 contributed to EndMT of HUVECs, JASPAR database was utilized to search potential interaction molecules of USF1. USP14 was predicted as a potential target of USF1 (Fig. 4A). To determine the direct interaction between USF1 and USP14, dual-luciferase reporter assay was performed. We found that overexpression of USF1 significantly enhanced the luciferase activity of wt-USP14, but not of mut-USP14 (Fig. 4B). ChIP assay further demonstrated that USP14 enrichment was markedly raised by immunoprecipitation with USF1 antibody in HUVECs (Fig. 4C). Therefore, USF1 could directly bind to USP14 promoter in HUVECs. Furthermore, overexpression of USF1 distinctly elevated USP14 expression, whereas depletion of USF1 led to the opposite result (Fig. 4D). USP14 is one of important deubiquitinating enzymes, which can eliminate ubiquitin before proteasome-mediated degradation (Fang et al. 2020). As predicted by Ubibrowser2.0 database, NLRC5 was a potential target protein of USP14 (Fig. 4E). In atherosclerotic tissue of *ApoE*^*–/–*^ mice fed with high fat, the deubiquitination of NLRC5 was increased, accompanied with the increase NLRC5 expression (Fig. S1). Notably, Co-IP assay showed that USP14 overexpression repressed the ubiquitin level of NLRC5 protein upon treatment with MG132, an inhibitor of proteasome (Fig. 4F). Furthermore, as expected, NLRC5 protein level was reduced in USP14-silenced, but enhanced in USP14-overexpressed HUVECs (Fig. 4G). Collectively, these results revealed that deubiquitinase USP14 was transcriptionally activated by USF1, which consequently increased NLRC5 protein level by inhibiting its ubiquitination and proteasomal degradation.

### USF1 activated USP14 to facilitate EndMT in vitro

**Fig. 5:**
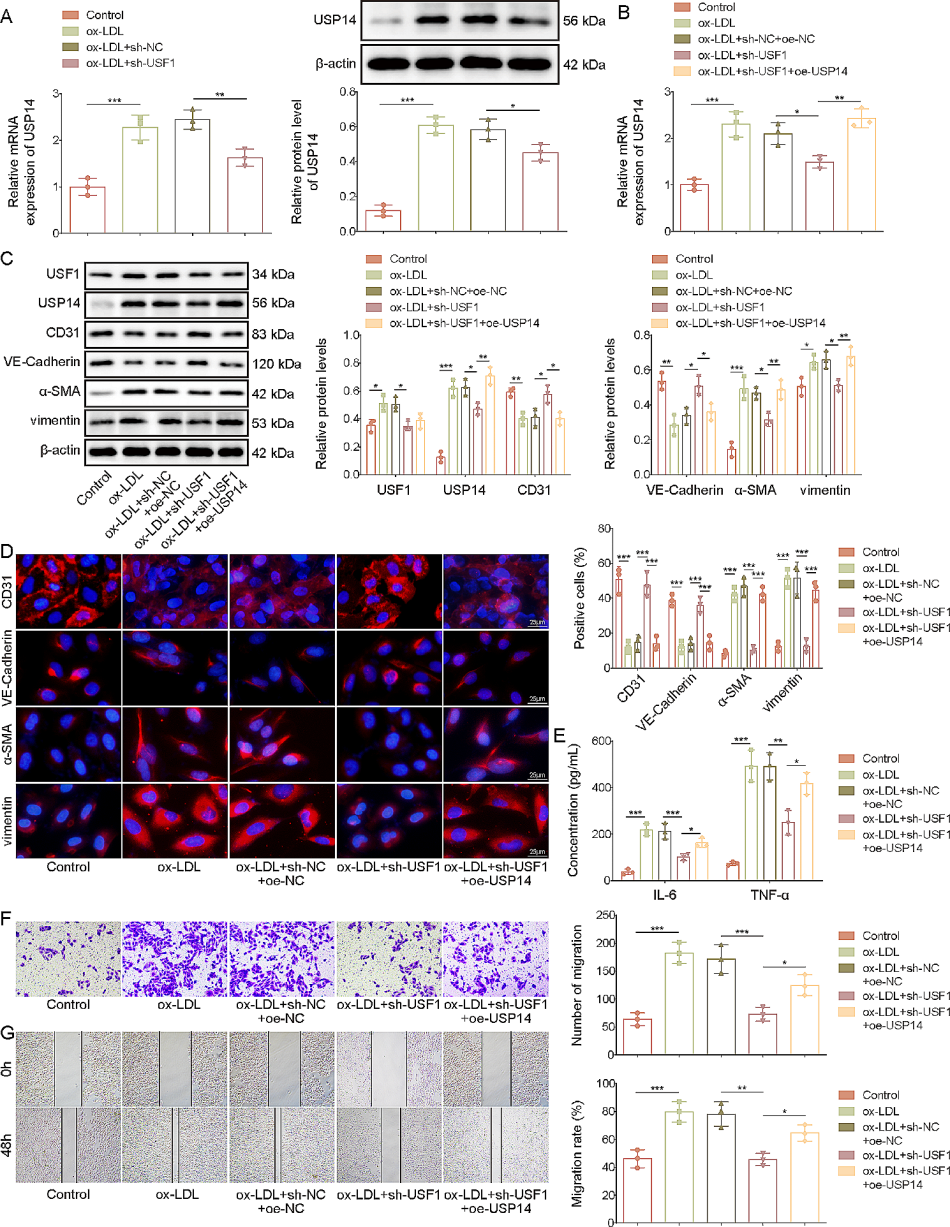
USF1 promoted EndMT through modulation of USP14. The HUVECs were transfected with sh-USF1 together with or without oe-USP14, followed by stimulation with 100 µg/mL ox-LDL for 24 h. (**A**)&(**B**) USP14 level was assessed by RT-qPCR and Western blotting. (**C**) Western blotting analysis of protein levels of USF1, USP14, EndMT markers CD31, VE-Cadherin, α-SMA, and vimentin. (**D**) Immunofluorescence staining of CD31, VE-Cadherin, α-SMA, and vimentin in HUVECs (scale bar = 25 μm). (**E**) Release of IL-6 and TNF-α from HUVECs was evaluated by ELISA kits. (**F**)&(**G**) Migration capacity of HUVECs was determined by transwell and scratch assays. *n* = 3. *p < 0.05, **p < 0.01, ***p < 0.001

To determine whether USP14 participated in USF1-mediated regulation in EndMT, sh-USF1 and oe-USP14 were co-transfected into HUVECs. As illustrated in Fig. 5A&B, USF1 silencing-mediated down-regulation of USP14 in ox-LDL-treated HUVECs. And the down-regulation of USP14 by USP1 knockdown was reversed by USP14 overexpression. Whereas overexpression of USP14 could not change the protein level of USF1 in sh-USF1-transfected HUVECs (Fig. 5C). Moreover, USF1 deficiency-mediated up-regulation of CD31 and VE-Cadherin, and down-regulation of α-SMA and vimentin in ox-LDL-stimulated HUVECs could be abolished after co-transfection with oe-USP14 (Fig. 5C**&D**). Notably, we observed that the decreased concentration of IL-6 and TNF-α induced by USF1 inhibition in ox-LDL-challenged HUVECs was partly recovered by USP14 overexpression (Fig. 5E). In addition, enforced USP14 expression partially restored the decreased cell migratory ability that was induced by USF1 suppression (Fig. 5F&G). Taken together, these results demonstrated that USF1 contributed to EndMT in HUVECs via regulation of USP14.

### USP14 silencing restrained EndMT through modulation of NLRC5/Smad2/3 pathway

**Fig. 6:**
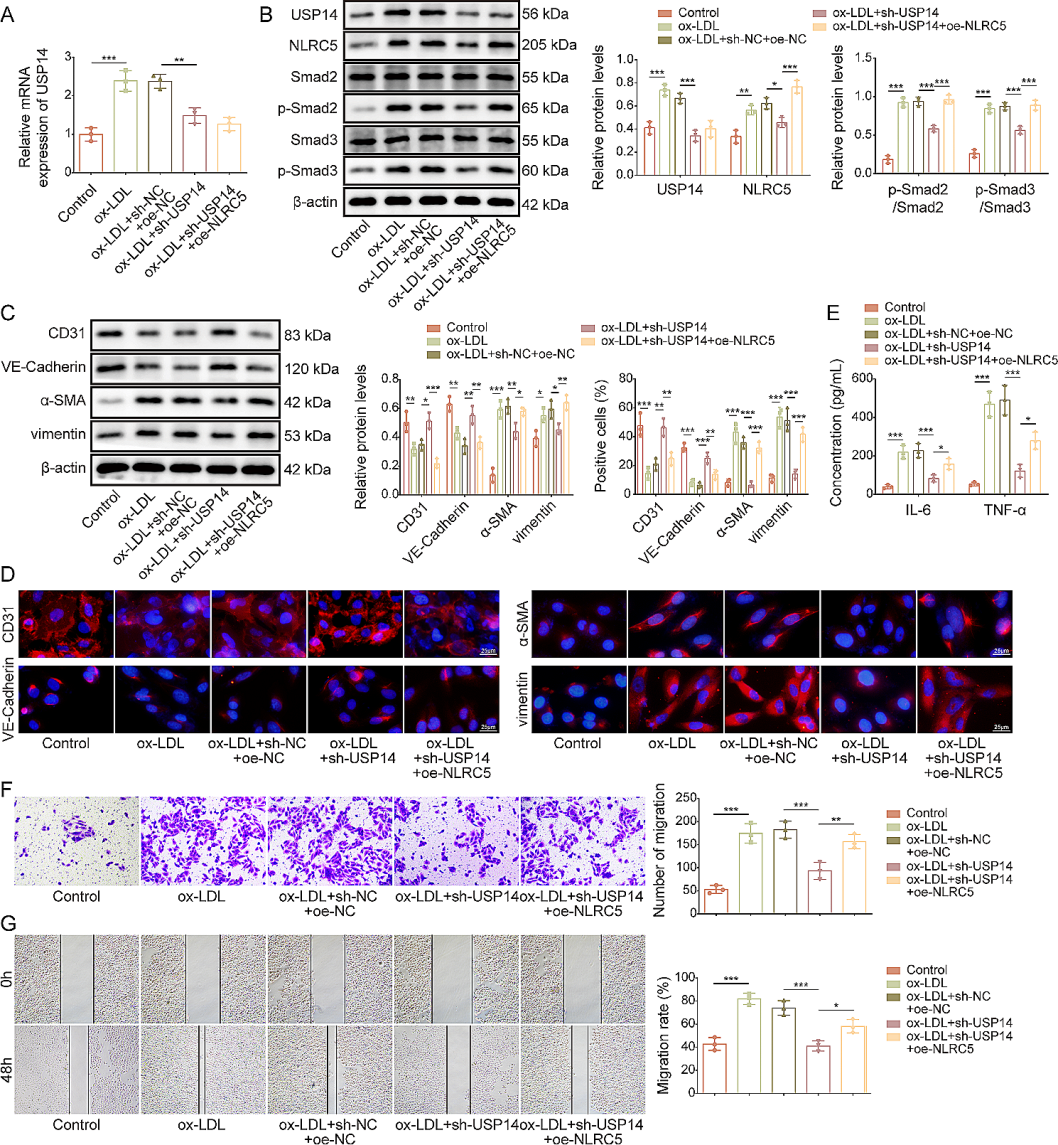
USP14 depletion repressed EndMT via inactivation of NLRC5/Smad2/3 pathway. The HUVECs were transfected with sh-USP14 combined with or without oe-NLRC5, followed by exposure to 100 µg/mL ox-LDL for 24 h. (**A**) RT-qPCR analysis of USP14 expression levels. (**B**) The abundance of USP14, NLRC5, Smad2, p-Smad2, Smad3, p-Smad3 was assessed by Western blotting. Expression of EndMT markers CD31, VE-Cadherin, α-SMA, and vimentin were measured by Western blotting (**C**) and immunofluorescence staining (**D**) (scale bar = 25 μm). (**E**) Concentrations of IL-6 and TNF-α produced by HUVECs were evaluated by ELISA kits. (**F**)&(**G**) Migration of HUVECs was evaluated by transwell and scratch assays. *n* = 3. *p < 0.05, **p < 0.01, ***p < 0.001

Next, we validated the involvement of NLRC5 in USP14-mediated biological function via rescue experiments. For this purpose, oe-NLRC5 was transfected into USP14-silenced HUVECs. NLRC5 overexpression restored the decreased expression of NLRC5, p-Smad2, and p-Smad3 in USP14-depleted cells, but not affected USP14 expression (Fig. 6A&B). Besides, NLRC5 overexpression also counteracted shUSP14-mediated up-regulation of CD31 and VE-Cadherin, and down-regulation of α-SMA and vimentin in ox-LDL-stimulated HUVECs (Fig. 6C&D). Moreover, the inhibitory effects of USP14 silencing on release of IL-6 and TNF-α and migration of ox-LDL-stimulated HUVECs were partly abrogated by NLRC5 overexpression (Fig. 6E-G). These findings suggested that USP14 depletion suppressed EndMT via inactivation of NLRC5/Smad2/3 pathway.

### Inactivating Smad2/3pathway ameliorated atherosclerosis via inhibiting EndMT in vivo

**Fig. 7:**
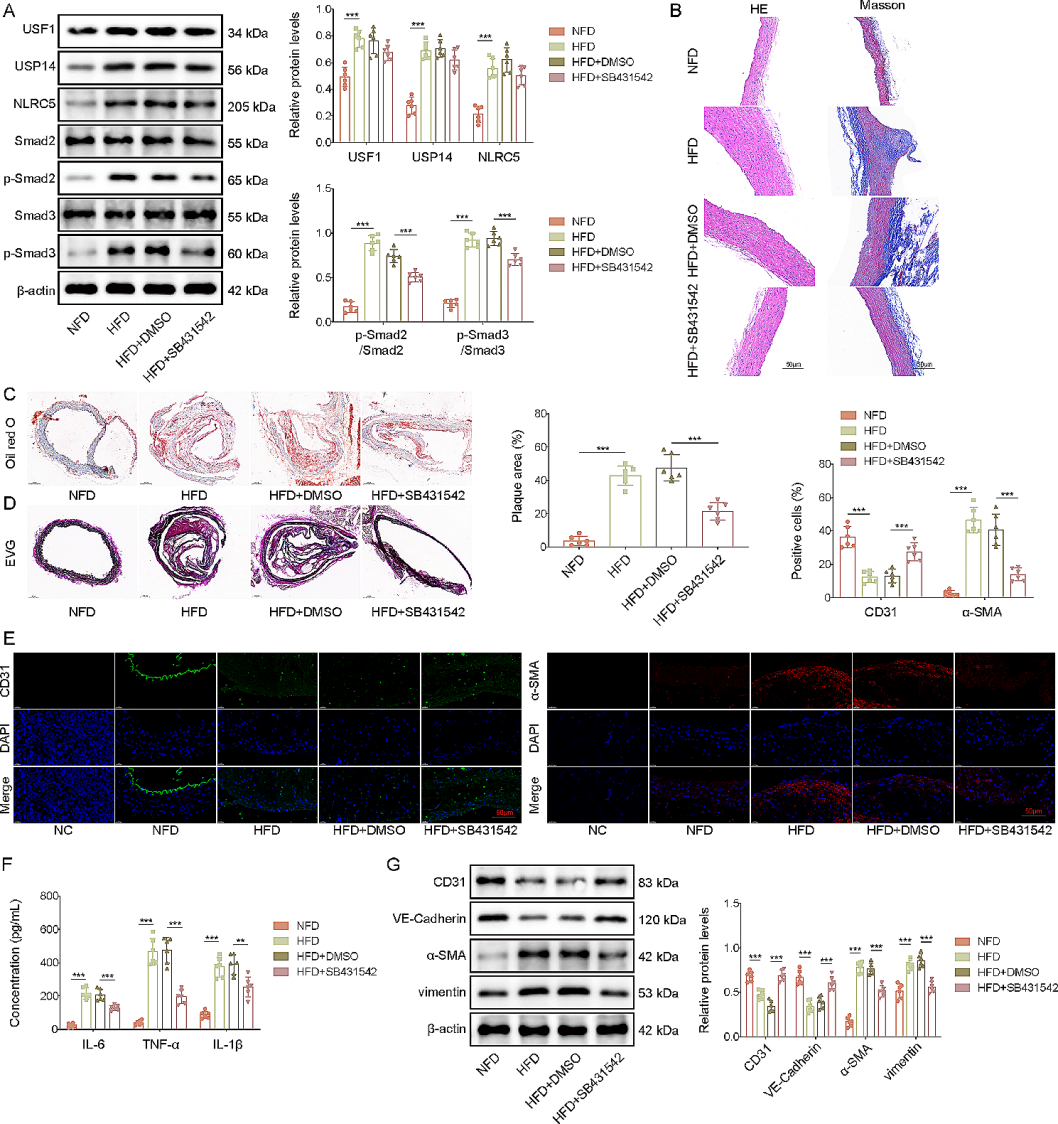
Inactivating Smad2/3 pathway ameliorated atherosclerosis via inhibiting EndMT *in vivo.* ApoE^–/–^ mice were fed with the Western high-fat diet for 3 months. SB431542 (4.2 mg/kg) was daily intraperitoneally injected into mice during experimental period. Normal chow-fed diet (NFD) represents normal mice. High-fat diet (HFD) represents atherosclerosis mice. (**A**) Western blotting analysis of USF1, USP14, NLRC5, Smad2, p-Smad2, Smad3, p-Smad3 levels in the aortic tissues. (**B**) The atheromatous plaque formation and collagen accumulation in the aortas was determined by HE and Masson staining (scale bar = 50 μm). (**C**) The aortic lipid deposition was assessed by Oil red O staining. (**D**) The morphological changes was evaluated by EVG staining. (**E**) Immunofluorescence staining of CD31 and α-SMA in the aortas of ApoE^–/–^ mice (scale bar = 50 μm). (**F**) The release of IL-6, TNF-α and IL-1β in serum was measured by ELISA kits. (**G**) Western blotting analysis of CD31, VE-Cadherin, α-SMA and vimentin levels in aortas. *n* = 6. **p < 0.01, ***p < 0.001

To verify the biological effects of USF1/NLRC5/Smad2/3 pathway on atherosclerosis progression in vivo, SB431542 (SMAD2/3 inhibitor) was intraperitoneally injected into *ApoE*^*–/–*^ mice, which were employed tofeed with high fat diet to establish an in vivo model for atherosclerosis. Treatment with SB431542 remarkably reduced p-Smad2 and p-Smad3 levels of atherosclerotic mice, but not affected USF1, USP14, and NLRC5 levels (Fig. 7A). Moreover, SB431542 treatment alleviated vascular stenosis and collagen deposition in the aortic tissues of atherosclerotic mice (Fig. 7B). As assessed by Oil red O and EVG staining, the aortic lipid deposition and pathological changes in the aortic tissues of *ApoE*^*–/–*^ mice could be effectively attenuated in SB431542 group (Fig. 7C&D). Moreover, CD31 was down-regulated, but α-SMA was up-regulated in atherosclerosis group, which could be reversed by SB431542 administration (Fig. 7E). Furthermore, SB431542 treatment reduced serum levels of IL-6, TNF-α and IL-1β in atherosclerotic mice (Fig. 7F). Western blotting showed that CD31 and VE-Cadherin expression was declined, but α-SMA and vimentin expression was raised in atherosclerosis model group; however, these alterations were abrogated by SB431542 (Fig. 7G). Collectively, these data demonstrated that activating Smad2/3 pathway was considered the downstream of USP14/NLRC5 axis to contribute to atherosclerosis progression by promoting EndMT in vivo.

## Discussion

Atherosclerosis has been recognized as a key contributor to cardiovascular and cerebrovascular disorders. Vascular endothelial dysfunction is one of notable characteristics of atherogenesis, which results in breakdown of endothelial barrier function and lipid deposition (Qin et al. 2020). Currently, EndMT plays pivotal roles in vascular endothelial dysfunction and facilitates plaque formation and its instability during pathobiological progression of atherosclerosis (Chen et al. 2015). EndMT has been recognized as a key driver of vascular inflammation (Liang et al. 2022; Chen et al. 2021a, b). More recently, EndMT has been suggested to have close correlation with atherosclerotic plaque instability (Li et al. 2017). These studies reinforce the fact that EndMT is of critical importance for governing vascular inflammation and plaque stability. Thus, the identification of new interventions for EndMT is urgently needed, which relies on illumination of its potential mechanisms. In this study, we found that USF1 expression was remarkably increased in atherosclerotic patients, and USF1 knockdown suppressed ox-LDL-induced EndMT in HUVECs. Mechanistically, USF1 functioned as a transcriptional activator of USP14 that caused de-ubiquitination of NLRC5, resulting in Smad2/3 pathway activation and EndMT induction in ox-LDL-exposed HUVECs. Finally, USF1 deficiency relieved atherosclerosis in ApoE^−/−^ mice through inhibiting EndMT.

Endothelial dysfunction is a pivotal process involved in the pathogenesis of atherosclerosis (Chen et al. 2019). EndMT was first defined by Leonard et al. during heart development (Eisenberg and Markwald 1995). During EndMT induction, endothelial marker proteins are lost, while mesenchymal marker proteins are acquired in endothelial cells. ox-LDL is considered as one of inducers of EndMT in endothelial dysfunction during atherosclerosis (Testai et al. 2021). In this work, endothelial dysfunction was induced by ox-LDL exposure in HUVECs. The cellular morphology of ox-LDL-stimulated HUVECs was changed from cobble-like into spindle-like cells, as well as down-regulation of endothelial markers and up-regulation of mesenchymal markers. Moreover, the migratory ability and pro-inflammatory cytokine release were significantly enhanced by ox-LDL. The in vivo results of ApoE^−/−^ mice were in line with the in vitro data. Therefore, EndMT was induced during atherosclerosis progression.

USF1 participates in the transcriptional modulation of multiple genes, which serves as a crucial regulator of a series of disorders, such as cancer (Sun et al. 2019), diabetes (Okamura et al. 2021), as well as atherosclerosis (Hoekstra et al. 2021). Li et al. found that USF1 was involved in lncRNA HIF1A-AS2-mediated modulation of ATF2 expression via direct binding to ATF2 promoter, which contributed to atherosclerotic inflammation (Li et al. 2020a, b). Consistently, we discovered that USF1 was up-regulated in atherosclerotic patients and experimental models. Interestingly, the regulation of USF1 in EndMT has been reported. For instance, USF1 silencing was identified to inhibit EndMT in melanoma cells (Ren et al. 2016). A recent study suggested that USF1 transcriptionally activated HAS2-AS1 to facilitate EndMT, thus promoting the invasion and migration of glioma cells (Wang et al. 2020a, b). In line with these observations, we found that USF1 knockdown repressed EndMT in atherosclerotic models. Our study firstly reported that USF1 facilitated atherosclerosis development via inducing EndMT.

Given that USF1 is a transcription factor, we further investigated its target gene in order to uncover its downstream modulatory mechanism. As analyzed by JASPAR database, USF1 possessed binding sites in the promoter of USP14. USP14 belongs to the DUB family that interplays with 26 S proteasome complex to enhance its de-ubiquitination activity (Hu et al. 2005). Aberrant expression of USP14 has close association with multiple diseases, including atherosclerosis (Liu et al. 2019). USP14 depletion was demonstrated to restrain foam cell formation through inhibiting CD36-mediated lipid uptake, which is considered as a therapeutic strategy for atherosclerosis (Zhang et al. 2020). Here, USP14 level was found to be positively correlated with USF1 level in the clinical samples of atherosclerosis. The low person value may be due to individual differences. However, enlarging sample size can reduce the impact of individual differences. In addition, USF1 directly bound to USP14 promoter to enhance its transcription and expression. Functionally, USP14 overexpression partly counteracted the inhibitory effect of USF1 deficiency on EndMT, thus other factors might also participate in the regulation of USF1 in EndMT. Thus, USF1 exacerbated atherosclerosis progression by transcriptional activation of USP14.

As a deubiquitinating enzyme, USP14 eliminates ubiquitin to cause de-ubiquitination and stabilization of its target proteins (Kim and Goldberg 2017). Here, NLRC5 was identified as a target protein that was de-ubiquitinated by USP14. NLRC5 is known as a pivotal mediator of inflammation (Wu et al. 2019). Ma et al. suggested that NLRC5 knockout repressed dermal fibrosis by inactivation of the Smad2/3 pathway (Ma et al. 2016). Specifically, NLRC5 deficiency suppressed high glucose-induced EndMT in endothelial cells through inactivation of Smad2/3 pathway (Wang et al. 2020a, b). In this study, we verified that USP14 enhanced NLRC5 protein level via de-ubiquitination of NLRC5. NLRC5 overexpression abolished sh-USP14-mediated inhibition in EndMT through activation of Smad2/3 pathway. These findings suggested that USP14-mediated de-ubiquitination of NLRC5 resulted in Smad2/3 pathway activation, which contributed to EndMT during atherosclerosis development.

EndMT is a gradual, reversible, and dynamic process. During EndMT, endothelial cells lose their characteristic markers, such as CD31, VE-cadherin, while gaining increased expression of mesenchymal markers, such as α-SMA, and vimentin. Notably, transforming growth factor β (TGF-β), a multifunctional cytokine is one of the best studied EndMT inducers (Yoshimatsu and Watabe 2011). Several groups have reported on signaling systems and molecules that induce EndMT, including Wnt/β-catenin and Notch signaling (Liu et al. 2014), as well as hypoxia (Zhang et al. 2018). In this study, the regulatory effect of USF1/USP14/NLRC5 axis on EndMT was comparable with these known regulators of EndMT, as evidenced by regulating CD31, VE-cadherin, α-SMA, and vimentin expression.

There are several limitations in this study. Firstly, it is hard to collect clinical tissue samples of atherosclerosis patients. Serum samples were detected in this study, which might not accurately reflect the actual condition in atherosclerotic tissues. Secondly, due to limited funds, we did not determine in vivo EndMT using lineage tracking mice. Third, how USF1 is regulated or activated in endothelial cells remains unclear. It has been documented that USF1 can be modulated by various miRNAs (Sun et al. 2019; Chen et al. 2021a, b; Li et al. 2019). Whether USF1 can be modulated by specific miRNA in endothelial cells deserves to be explored. In our future study, these issues need to be addressed.

## Conclusion

Taken together, we uncover a USF1/USP14/NLRC5/Smad2/3 axis that drives atherosclerosis progression via inducing EndMT. Our observations suggest USF1 as a prognostic marker, as well as a novel potential therapeutic target for atherosclerosis.

### Electronic supplementary material

Below is the link to the electronic supplementary material.

Supplementary Material 1

Supplementary Material 2

## Data Availability

The datasets generated during and/or analysed during the current study are available from the corresponding author on reasonable request.

## Abbreviations

EndMT: Endothelial to mesenchymal transition
USF1: Upstream stimulating factor 1
USP14: Ubiquitin specific protease
NLRC: 5NLR Family CARD Domain Containing 5
HUVECs: Human umbilical vein endothelial cells
sh-USF1: shRNA targeting USF1
oe-USF1: USF1 overexpression plasmid
RT-qPCR: Quantitative real time polymerase chain reaction
HE: Hematoxylin and eosin
ELISA: Enzyme linked immunosorbent assay
Co-IP: Co-immunoprecipitation
ChIP: Chromatin immunoprecipitation
EVG: Verhoeff-Van Gieson
SD: Standard deviation
ANOVA: One-way analysis of variance
