# Supplementary figures and images for: USF1 transcriptionally activates USP14 to drive atherosclerosis by promoting EndMT through NLRC5/Smad2/3 axis
Source: Mol Med. 2024 Feb 29;30:32. doi: 10.1186/s10020-024-00798-8 (PMC10905873; doi:10.1186/s10020-024-00798-8)

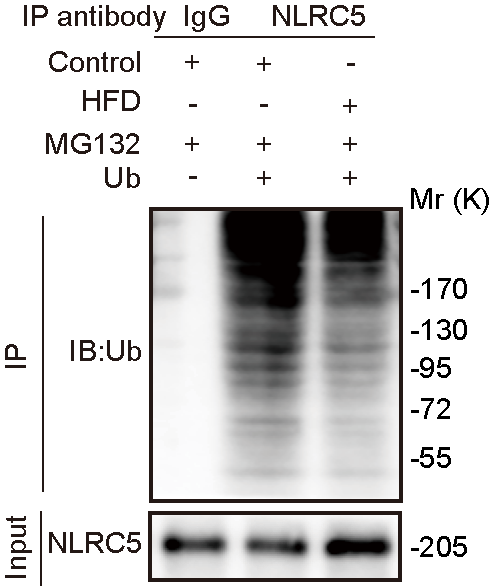

Supplement: Supplementary file 1 — Supplementary Material 1 [file 10020_2024_798_MOESM1_ESM.tif]
