# Supplementary material for: USF1 transcriptionally activates USP14 to drive atherosclerosis by promoting EndMT through NLRC5/Smad2/3 axis
Source: Mol Med. 2024 Feb 29;30:32. doi: 10.1186/s10020-024-00798-8 (PMC10905873; doi:10.1186/s10020-024-00798-8)
Supplement: Supplementary file 2 — Supplementary Material 2 [file 10020_2024_798_MOESM2_ESM.docx]

Reviewer reports:

Reviewer #1: In the paper entitled "USF1 transcriptionally activates USP14 to drive atherosclerosis by promoting EndMT" and authored by Zhiwen Zhang and collaborators, the authors described a new mechanism possibly involved in EndoMT that would occur during Atherosclerosis. While there in vitro data are pretty convincing, i.e. Oxidized LDL would promote EndoMT via upregulation of USF1 transcription factors in endothelial cells. Mechanistically, USF1 would trans-activate USP14 promoter leading to USP14 upregulation and decreased NLRC5 degradation. This is associated with increased Smad2 and Smad3 phosphorylation. On the contrary, the in vivo data presented in this manuscript are far from being convincing.

As an example the CD31 staining shown on Figure 1B, shows CD31 is expressed by the media but not the endothelium in a healthy aorta??? Also SMA is barely expressed in the media of a healthy aorta???

See my comments below

Fig 1: What could be the sources of USF1 and USP14 mRNA in the serum of patients? micro particles?

**Response:** Thank you for your insightful question. A small number of detached vascular endothelial cells are in the serum^1^. During atherosclerotic plaque formation, the intima of the aorta is severely damaged and the endothelial cells are detached. In addition, serum-derived exosomes contain molecules from their paternal cells, including some proteins, nucleic acids, and lipids^2^. Therefore, the detached vascular endothelial cells and serum-derived exosomes might be the sources of USF1 and USP14 mRNA in the serum of patients.

**References:**

1.	Rakic M, Persic V, Kehler T, et al. Possible role of circulating endothelial cells in patients after acute myocardial infarction. Med Hypotheses 2018;117: 42-46.

2.	Alejandre Gonzalez AG, Ortiz-Lazareno PC, Solorzano-Ibarra F, et al. A Modified Method for the Quantification of Immune Checkpoint Ligands on Exosomes from Human Serum using Flow Cytometry. Technol Cancer Res Treat 2023;22: 15330338221150324.

Fig 2A: SMA upregulation could be due to increased SMCs proliferation. SMA expression by endothelial cells is more likely negligible when using total aorta extracts.

**Response:** Thank you for your comment. During AS development, hyper-proliferation of SMCs may lead to α-SMA up-regulation. The mRNA level of α-SMA in total aorta extracts (containing both SMCs and endothelial cells) was detected in **Fig. 2A**. Therefore, the result indicated that α-SMA level was higher in AS model as compared with normal group. Here, we did not determine α-SMA expression in endothelial cells.

Fig 2B: The CD31 staining is rather not specific so is the SMA staining since at least in the healthy aorta; anti-CD31 antibodies should stain only the endothelium and SMA, smooth muscle cells. For instance both CD31 staining and SMA staining appears to be located in the media, and the healthy aorta does not seem to include SMA positive smooth muscle cells????

**Response:** Thank you for your comment. As demonstrated by Tober et al^3^, CD31 can be widely expressed in the whole aorta. Our results also showed that CD31 and α-SMA were widely expressed in aorta (**Fig. 2B and Fig. 7E**). We are sorry about that the poor picture quality causes your confusion. We have reperformed the immunofluorescent staining of CD31 and α-SMA. Please see the revised **Fig. 2B, 7E** for detail.

**Reference:**

3.	Tober J, Yzaguirre AD, Piwarzyk E, Speck NA. Distinct temporal requirements for Runx1 in hematopoietic progenitors and stem cells. Development 2013;140(18): 3765-76.

Fig 2 general comment: In vivo EndoMT should be demonstrated using lineage tracking mice, other wise it is not possible to make any conclusions

**Response:** We appreciate your advice. As previously described^4^, we evaluated *in vivo* EndMT via detection of CD31, α-SMA, VE-cadherin and vimentin levels. Due to limited funds, we will determine *in vivo* EndoMT using lineage tracking mice in our future study. We have discussed this issue as a limitation of our study. Please see the Discussion section (the last paragraph) for detail. We really appreciate your understanding.

**Reference:**

4.	Diao H, Cheng J, Huang X, et al. The Chinese medicine Fufang Zhenzhu Tiaozhi capsule protects against atherosclerosis by suppressing EndMT via modulating Akt1/beta-catenin signaling pathway. J Ethnopharmacol 2022;293: 115261.

Fig 7A: The role of Smad2 and 3 phosphorylation in mediating USF1-induced endoMT should be demonstrated using inhibitors.

**Response:** Thank you for your advice. SB431542(SMAD2/3 inhibitor) has been adopted according to your suggestion. We found that treatment with SB431542 reversed AS progression, but did not affect USF1, USP14, and NLRC5 expression. Please see the revised **Fig. 7** and Result section (the seventh paragraph) for detail.

Fig 7C-G: It is not surprising administration of USF1 shRNA prevent the development of atherosclerosis, since USF1 deficient mice were previously shown to be protected from atherosclerosis (doi: 10.1126/scitranslmed.aad0015). However, it does not mean USF1 deficiency prevents endoMT in vivo. Once again the staining shown Fig 7E are more than doubtful.

**Response:** We really appreciate your comment. Our study for the first time demonstrated the role of USF1 in endoMT during the development of atherosclerosis, which is the novelty of this study. We have replaced the images of immunofluorescent staining for CD31 and α-SMA in **Fig. 2B, 7E**.

Reviewer #2: First of all, congratulations to the authors for this exciting study. EndMT is a very complex topic and this study is essential for the field. I want to suggest some changes and request a few more information. Please see below:

1-) It would be helpful to have more information about the patients with atherosclerosis and healthy controls to make sure that other parameters did not alter the USF1 and USP14 levels in serum. How did you guys select the patients and controls? How was the presence of atheroma observed? Were they matched for age, sex... in the controls and atherosclerosis group?

**Response:** Thanks so much for your suggestion. As you suggested, we have added a table with the characteristics of the patients, and analyzed their association with AS. The result showed that AS was closely correlated with body mass index, but not associated with age, gender, history of smoking, diabetics, or chronic kidney disease. The AS patients and controls were selected according to the diagnosis of atherosclerosis based on the value of the carotid intima-media thickness (CIMT) of the common carotid artery. They matched for age, sex in the controls and atherosclerosis group.

Please see **Table 1**, Method section (Clinical samples) and result section (the first paragraph) for detail.

2-) About the mouse model of atherosclerosis. Was it the same diet time in figure 2 and figure 7? Did you guys saw lipid deposition in the figure 2 by Oil red O staining? It should be included as well.

**Response:** Thank you for your questions. It was the same diet time in **Fig. 2** and **Fig. 7**. Oil red O staining has been conducted in **Fig. 2**. As shown in **Fig. 2C**, lipid deposition was found in AS model group. Please see the Result section (the second paragraph) for detail.

3-) There is no information about the number of experiments and animals per group. It must be included in all Figure legends. If it's possible, show the points for each sample in the graph bar.

**Response:** Thank you for your valuable suggestion. For *in vitro* assays, n = 3; for in vivo assays, n = 6; for clinical samples, n= 50. We have explained this in all Figure legends. The results are presented by dot values in all the graphics. Please see the Figure legends section and Figures for detail.

4-) Fig. 4F, I can't fully understand which band in anti-ha you are talking about. Could you make it more clear? Please include the molecular weight too.

**Response:** Thanks so much for your suggestion. The molecular weight for all proteins has been provided. For **Fig. 4F**, it should be anti-Ubiquitin, but not anti-HA. We are sorry for our carefulness. Please see the revised **Fig. 4F** for detail.

5-) Fig. 5E, you can't say that the overexpression of USF1 recovered IL-6 and TNFa levels. It decreased the levels but it's still higher than the control. Probably other factors regulate this.

**Response:** Thank you for your insightful comment. We have changed the description that “the decreased IL-6 and TNF-α concentration observed under USF1 inhibition in ox-LDL-challenged HUVECs was partly recovered by USP14 overexpression”. This might be because that other factors might also participate in the regulation of USF1, which has been discussed in the Discussion section (the fourth paragraph).

Reviewer #3: Is an interesting manuscript to read where is described the regulation of USF1/USP14/NLRC5 axis in EndMT associated with atherosclerosis. The methodology is integrated by in-vivo and in-vitro models. The author's strategy was knocking down the expression USF1 and describe the changes under USP14 silencing or overexpression. USF1 has been related before with lipids regulation and inflammation, however never been described in EndMT-related atherosclerosis.

See below my comments and suggestions.

1.	Please declare what was the strategy to avoid the analysis bias in the immunohistochemistry and the immunofluorescence analysis.

**Response:** We appreciate your suggestion. To avoid bias, quantitative analysis has been performed for immunofluorescence staining using the Image J software. Please see the revised **Fig. 2B&G, 3C, 5D, 6D, 7C, 7E** for detail.

2.	In the scratch assay, add the pipette tip size (200 or 500µl) used.

**Response:** Thanks so much for your suggestion. The pipette tip (500 µL) was used in the scratch assay. We have explained this in the Method section (Scratch assay).

3.	In the analysis in serum patients. Please consider in the analysis the gender difference and age in, and if possible, re-analysis the data considering differences between women and men. Be aware that USF1 had reported being subject to gender differences (Fan YM, et al. Sci Rep. 2014, PMID: 24722012). Integrated a paragraph in the discussion about gender tendency if the analysis shows significant differences. Add a table with the characteristics of the patients (age, and gender as preferences).

**Response:** We really appreciate your valuable suggestion. The study by Fan YM et mainly focused on the effects of USF1 gene allelic variants on lipid metabolism in a gender-dependent manner, and the research emphases are different from this study. As you suggested, we have added a table with the characteristics of the patients, and analyzed their association with AS. The result showed that AS was closely correlated with body mass index, but not associated with age, gender, history of smoking, diabetics, or chronic kidney disease. Please see **Table 1** and result section (the first paragraph) for detail.

4.	In Figure 1B, even the analysis demonstrated a significant association. Person value is low, please discuss it.

**Response:** Thank you for your valuable suggestion. The low person value in **Fig. 1B** may be due to individual differences. However, enlarging sample size can reduce the impact of individual differences. We have discussed this issue in the Discussion section (the fourth paragraph).

5.	Quantify the immunofluorescence and add the graphics. If the images are not quantified are subject to bias.

**Response:** Thanks so much for your suggestion. The immunofluorescence has been quantified. Please see the revised **Fig. 2B&G, 3C, 5D, 6D, and 7E** for detail.

6.	In the graphics, I advise change the way the results are presented. Add dots values in the graphics where the reader can see where the results values are. That shows transparency in the results and reliability.

**Response:** We appreciate your suggestion. The results are presented by dot values in all the graphics. Please see the revised figures for detail.

7.	In the in-vitro assay, establish if were technical o biological replicates.

**Response:** Thank you for your valuable suggestion. For *in vitro* assay, both technical replicates and biological replicates were performed. For *in vivo* assay, biological replicates were performed. We have explained this in the Method section (Statistical analysis).

8.	In Figure 4F, Anti-H is not clear. That figure is subject to bias. Please consider modify.

**Response:** Thank you for your suggestion. We have re-performed this experiment. For **Fig. 4F**, it should be anti-Ubiquitin, but not anti-HA. We are sorry for our carefulness. Please see the revised **Fig. 4F** for detail.

9.	Add a paragraph discussing the study limitations.

**Response:** We really appreciate your valuable suggestion. Study limitations have been discussed in the discussion section (the last paragraph).

Reviewer #4: Here the authors describe the role of USF1 in atherosclerosis. The authors claim that USF1 transcriptionally activates USP14, which promotes deubiquitination and up-regulation of NLRC5, leading to activation of Smad2/3 pathway and induction of EndMT in endothelial cells. They provide evidence from clinical samples, animal models and cell experiments to support their hypothesis. They conclude that USF1/USP14/NLRC5 axis contributes to atherosclerosis development via promoting EndMT and suggest it as a potential therapeutic target.

Major points:

The authors address a relevant and important topic in cardiovascular research.

The authors provide novel insights into the molecular mechanism of EndMT in atherosclerosis.

The authors use multiple approaches and techniques to validate their findings, as well as appropriate statistical tests.

The authors identify a potential therapeutic target for atherosclerosis prevention and treatment.

**Response**: We really appreciate your recognition of our study.

Minor points:

The authors do not explain how USF1 is regulated or activated in endothelial cells.

The authors do not show the functional consequences of EndMT on vascular inflammation and plaque stability.

The authors do not compare the effects of USF1/USP14/NLRC5 axis with other known regulators of EndMT.

These different points could be commented in the discussion.

**Response:** Thanks so much for your advices. How USF1 is regulated or activated in endothelial cells remains unclear. It has been documented that USF1 can be modulated by various miRNAs. Whether USF1 can be modulated by specific miRNA in endothelial cells deserves to be explored. We have discussed this in the last paragraph of Discussion section. The functional consequences of EndMT on vascular inflammation and plaque stability (the first paragraph), the effects of USF1/USP14/NLRC5 axis in comparison with other known regulators of EndMT (the second paragraph from bottom) have been discussed in the Discussion section. Please see the revised manuscript for detail.

Some minor typos:

Line 1: "atherosclerotic cardiovascular disease (ASCVD)" should be capitalized as "Atherosclerotic Cardiovascular Disease (ASCVD)".

Line 2: "endothelial-to-mesenchymal transition (EndMT)" should be capitalized as "Endothelial-to-Mesenchymal Transition (EndMT)".

**Response:** Thank you for your suggestion. We have capitalized "Endothelial-to-Mesenchymal Transition (EndMT)" as you suggested, however, there is no words "atherosclerotic cardiovascular disease (ASCVD)" in the manuscript.

Reviewer #5: Zhang and colleagues investigated the role of upstream stimulating factor 1 (USF1) in endothelial mesenchymal transition (EndMT) in in vivo and in vitro models of atherosclerosis. While the study in general is well performed, several inconsistencies decrease the overall significance of the findings as detailed below.

1.	Figure 1. Authors need to include inflammatory marker measurements from serum samples

**Response:** We really appreciate your valuable advice. The serum levels of inflammatory markers IL-6 and TNF-α were detected by ELISA. According to the results, the serum levels of IL-6 and TNF-α were significantly higher in patients with atherosclerosis as compared with normal volunteers. Please see the revised **Fig. 1C** and Result section (the first paragraph) for detail.

2.	Results, Page#11, line#3, authors suggest performing RT-qPCR for USF1 and USP14. I could not find procedure for RNA isolation from human serum in the methods section.

**Response:** Thanks so much for your suggestion. USF1 and USP14 mRNA levels were detected by RT-qPCR. The results showed that USF1 and USP14 mRNA levels were elevated in the aortas of HFD-induced mice. Total RNA was isolated from human serum samples using TRIzol reagent (Thermo Fisher). Please see the revised **Fig. 2A**, Result section (the second paragraph), and Method section (RT-qPCR) for detail.

3.	Figure #2 and figure #7, There is a need for inclusion of quantitation of stenosis/palque.

**Response:** We really appreciate your valuable advice. The plaque area was quantified according a previous study. Please see the revised **Fig. 2C** and **Fig. 7C** for detail.

4.	Authors use HUVECs to model EndMT during atherosclerosis. This is not an ideal model and does not reflect an in vivo situation as veins rarely display atherosclerosis and HUVEC and HAECs display different response to biophysical and biochemical stimuli.

**Response:** Thank you for your insightful comment. ox-LDL-induced HUVECs have been used as an *in vitro* model of EndMT during atherosclerosis in a great number of studies^4-10^. According to these studies, we established the EndMT model by treatment with ox-LDL in HUVECs for 24 h. ox-LDL-induced human aortic endothelial cells (HAECs) have also been used to establish EndMT model^9^^,^ ^11^. As compared with HUVECs, HAECs are more sensitive to ox-LDL. Generally, HAECs are treated with 40-50 μg/mL ox-LDL for 4 or 8 h, while HUVECs are treated with 100 μg/mL ox-LDL for 24 h. Therefore, both HUVECs and HAECs can use to establish EndMT model during atherosclerosis.

**References:**

4.	Diao H, Cheng J, Huang X, et al. The Chinese medicine Fufang Zhenzhu Tiaozhi capsule protects against atherosclerosis by suppressing EndMT via modulating Akt1/beta-catenin signaling pathway. J Ethnopharmacol 2022;293: 115261.

5.	Gong L, Lei Y, Liu Y, et al. Vaccarin prevents ox-LDL-induced HUVEC EndMT, inflammation and apoptosis by suppressing ROS/p38 MAPK signaling. Am J Transl Res 2019;11(4): 2140-54.

6.	Li H, Zhao Q, Chang L, et al. LncRNA MALAT1 modulates ox-LDL induced EndMT through the Wnt/beta-catenin signaling pathway. Lipids Health Dis 2019;18(1): 62.

7.	Bai Y, Liu X, Chen Q, Chen T, Jiang N, Guo Z. Myricetin ameliorates ox-LDL-induced HUVECs apoptosis and inflammation via lncRNA GAS5 upregulating the expression of miR-29a-3p. Sci Rep 2021;11(1): 19637.

8.	Zhang X, Li Z, Liu X, et al. ZPI prevents ox-LDL-mediated endothelial injury leading to inhibition of EndMT, inflammation, apoptosis, and oxidative stress through activating Pi3k/Akt signal pathway. Drug Dev Res 2022;83(5): 1212-25.

9.	Mao Y, Jiang L. MiR-200c-3p promotes ox-LDL-induced endothelial to mesenchymal transition in human umbilical vein endothelial cells through SMAD7/YAP pathway. J Physiol Sci 2021;71(1): 30.

10.	Gao F, Zhao Y, Zhang B, et al. SESN1 attenuates the Ox‑LDL‑induced inflammation, apoptosis and endothelial‑mesenchymal transition of human umbilical vein endothelial cells by regulating AMPK/SIRT1/LOX1 signaling. Mol Med Rep 2022;25(5).

11.	Su Q, Sun Y, Ye Z, Yang H, Li L. Oxidized low density lipoprotein induces endothelial-to-mesenchymal transition by stabilizing Snail in human aortic endothelial cells. Biomed Pharmacother 2018;106: 1720-26.

5.	The rationale for 24h for ox-LDL induced EndMT is not clear. Did the authors perform any time series experiment to arrive at this time point ?

**Response:** We really appreciate your advice. According to the previous studies^4^^,^ ^5^^,^ ^12^, EndMT was induced by treatment with ox-LDL for 24h. We have explained this in the Method section (Cell culture and treatment).

**References:**

4.	Diao H, Cheng J, Huang X, et al. The Chinese medicine Fufang Zhenzhu Tiaozhi capsule protects against atherosclerosis by suppressing EndMT via modulating Akt1/beta-catenin signaling pathway. J Ethnopharmacol 2022;293: 115261.

5.	Gong L, Lei Y, Liu Y, et al. Vaccarin prevents ox-LDL-induced HUVEC EndMT, inflammation and apoptosis by suppressing ROS/p38 MAPK signaling. Am J Transl Res 2019;11(4): 2140-54.

12.	Qiu Y, Du B, Xie F, et al. Vaccarin attenuates high glucose-induced human EA*hy926 endothelial cell injury through inhibition of Notch signaling. Mol Med Rep 2016;13(3): 2143-50.

6.	What was the rationale to use GAPDH for RT-qPCR normalization, whereas use b-actin for western blot assays.

**Response:** Thank you for your question. Both GAPDH and β-actin are commonly used reference genes for RT-qPCR and western blot assays. Thus, either one is suitable for RT-qPCR and western blot assays.

7.	Figure 2, panel B and Figure 7, panel E. Authors need to improve the quality of the CD-31 and SMA stainings. Additionally, please include antibody negative controls.

**Response:** We really appreciate your advice. We have re-performed immunofluorescence staining for CD31 and SMA to improve the image quality. In addition, CD31 immunofluorescence staining in cervical squamous cell carcinoma cells and SMA immunofluorescence staining in adipocytes were used as negative controls for CD31 and SMA immunofluorescence staining, respectively. Please see the revised **Fig. 2B** and **Fig. 7E** for detail.

8.	Abstract, Page#2, line #20, please modify 'corruption of elastin'.

**Response:** Thanks so much for your suggestion. We have changed 'corruption of elastin' to ' morphological changes ' in Abstract. Please see the revised manuscript for detail.

Reviewer #6: Zhang et al demonstrated that USF1 drives atherosclerosis progression by transcriptionally activating USP14, and EndMT through NLRC5/ Smad2/Smad3 pathway.

Specific comments:

1.)	In Figure legend 2, 7 no description is written for sham and AS. What is sham and AS denote? Sham should be replaced by term normal chow-fed diet (NC) and AS should be replaced by term HFD (High-fat diet). Sham cannot be used as it is not a surgery mouse.

**Response:** Thank you for your suggestion. Sham is replaced by normal chow-fed diet (NFD) and AS is replaced by HFD (High-fat diet) in **Fig.2 and Fig. 7**, which have been described in Figure legend 2, 7. Please see the revised figures and Figure legend section for detail.

2.)	Deubiquitination of NLRC5 by USP14 is not shown for atherosclerotic tissue.

**Response:** We really appreciate your valuable advice. Since it is hard to collect clinical AS samples from patients, we have evaluated the ubiquitin level of NLRC5 in the aortas of HFD-induced mice and NC mice. For **Fig. S1**, in atherosclerotic tissue of *ApoE^–/–^* mice fed with high fat, the deubiquitination of NLRC5 was increased, accompanied with the increase NLRC5 expression. Please see the revised **Fig. S1** for detail.

3.)	Please correct for grammer e.g. in ChIP assay line 58, was performed to "broke" should be was performed to "break". In conclusion, line number 3 that "drove" atherosclerosis should be "drive" atherosclerosis.

**Response:** Thanks so much for your suggestion. We have corrected the grammatical mistakes throughout the manuscript. Please see the revised manuscript for detail.
